# Mode of administration influences plasma levels of active *Centella asiatica* compounds in 5xFAD mice while markers of neuroinflammation remain unaltered

**DOI:** 10.3389/fnins.2024.1277626

**Published:** 2024-03-25

**Authors:** Alex B. Speers, Kirsten M. Wright, Mikah S. Brandes, Nareg Kedjejian, Donald G. Matthews, Maya Caruso, Christopher J. Harris, Seiji Koike, Thuan Nguyen, Joseph F. Quinn, Amala Soumyanath, Nora E. Gray

**Affiliations:** ^1^BENFRA Botanical Dietary Supplements Research Center, Oregon Health & Science University, Portland, OR, United States; ^2^Department of Neurology, Oregon Health & Science University, Portland, OR, United States; ^3^School of Public Health, Oregon Health & Science University-Portland State University, Portland, OR, United States; ^4^Parkinson’s Disease Research Education and Clinical Care Center, Veterans’ Administration Portland Health Care System, Portland, OR, United States

**Keywords:** *Centella asiatica*, Alzheimer’s disease, neuroinflammation, 5xFAD, triterpenes, caffeoylquinic acids

## Abstract

**Introduction:**

A water extract of *Centella asiatica* (L.) Urban [Apiaceae] (CAW) has demonstrated cognitive-enhancing effects in mouse models of Alzheimer’s disease and aging, the magnitude of which is influenced by whether CAW is delivered in the drinking water or the diet. These cognitive benefits are accompanied by improvements in oxidative stress and mitochondrial function in the brain, two pathways related to the neuroinflammatory response. The effect of CAW on neuroinflammation, however, has not been directly studied. Here, we investigated the effect of CAW on neuroinflammation in 5xFAD mice and compared plasma levels of CAW’s active compounds following two modes of CAW administration.

**Methods:**

Eight-to-nine-month-old male and female 5xFAD mice and their wild-type littermates were administered CAW in their diet or drinking water (0 or 1,000 mg/kg/day) for five weeks. Immunohistochemistry was performed for β-amyloid (Aβ), glial fibrillary acidic protein (GFAP), and *Griffonia simplicifolia* lectin I (GSL I) in the cortex and hippocampus. Gene expression of inflammatory mediators (IL-6, TNFα, IL-1β, TREM2, AIF1, CX3CR1, CX3CL1, CD36, C3AR1, RAGE, CCR6, CD3E) was measured in the deep grey matter.

**Results:**

CAW decreased cortical Aβ plaque burden in female 5xFAD mice administered CAW in the drinking water but had no effect on Aβ plaques in other treatment groups. CAW did not impact elevated levels of GFAP or GSL I in 5xFAD mice, regardless of sex, brain region, or mode of CAW administration. In the deep grey matter, CAW increased C3AR1 expression in 5xFAD females administered CAW in the drinking water and decreased IL-1β expression in 5xFAD males administered CAW in the diet. CAW had no effect, however, on gene expression levels of any other inflammatory mediator in the deep grey, for either sex or mode of CAW administration. Mice administered CAW in the drinking water versus the diet had significantly higher plasma levels of CAW compounds.

**Discussion:**

CAW had little impact on the neuroinflammatory markers selected for evaluation in the present study, suggesting that the cognitive benefits of CAW may not be mediated by an anti-inflammatory effect or that additional molecular markers are needed to fully characterize the effect of CAW on neuroinflammation.

## 1 Introduction

Alzheimer’s disease (AD), a progressive neurodegenerative disease, is the most common cause of dementia and the fifth-leading cause of death among those aged 65 years and older (Wong, 2020). While the incidence rate of AD has been declining in recent decades, the number of people with AD is expected to more than double by 2060 as a result of a rapidly aging US population (Alzheimer's Association, 2022). In addition to the burden this will place on individuals with AD and their families, the rise in AD cases in the coming decades will also increasingly tax the US healthcare system, with costs for AD care expected to total more than $1 trillion by 2050 (Wong, 2020). A major challenge has been the lack of available treatment options. Until recently, the US Food and Drug Administration (FDA) had only approved five drugs for AD, all of which treat AD-related cognitive symptoms without targeting the underlying brain pathology (Alzheimer's Association, 2022). Two new monoclonal antibodies however, aducanumab and lecanemab, target β-amyloid (Aβ) plaque formation and have recently been approved by the FDA for use in early AD (Alzheimer's Association, 2022). A third monoclonal antibody, donanemab, has also shown benefits in early AD in a recently published phase III trial (Sims et al., 2023). These drugs represent a new class of drug therapy for AD, targeting the hallmark neuropathological features of AD (i.e., deposition of Aβ plaques). However, focusing on Aβ plaques exclusively may not be sufficient to impede the progression of the disease.

Emerging theories of AD pathogenesis offer new avenues for drug development based around fundamental biological processes that may be disrupted early on in the disease process. Neuroinflammation, for example, is now thought to play a key role in AD pathogenesis (Ardura-Fabregat et al., 2017; Onyango et al., 2021), beginning decades before the clinical onset of AD (Hampel et al., 2020). The neuroinflammatory pathway is mediated by several cell types in the brain (e.g., microglia and astrocytes) (Leng and Edison, 2021; Onyango et al., 2021) and has crosstalk with other pathways relevant to disease progression, including those involved in mitochondrial dysfunction and oxidative stress (Picca et al., 2020; Onyango et al., 2021). Given the multifaceted nature of AD and its underlying pathophysiology, there is growing interest in developing therapies that can simultaneously target the multiple biological processes disrupted in AD (Ju and Tam, 2022).

Herbal therapies, which contain a multitude of phytochemical components with various biological targets, offer one potential avenue in the development of multi-mechanistic agents for AD (Gregory et al., 2021). The herb *Centella asiatica* (L.) Urban [Apiaceae], commonly known in the US as gotu kola, is one such example*. Centella asiatica* is a plant traditionally used in Chinese and Ayurvedic medicine for its reputed effects on brain health (Gray et al., 2017), and is commercially available in various botanical dietary supplements. Pre-clinical and clinical studies have provided early evidence of its cognitive-enhancing effects (Gray et al., 2017), suggesting a potential therapeutic role for AD, but our understanding of the mechanisms underlying these cognitive effects is still evolving.

Our group has studied a water extract *of Centella asiatica* (CAW) in mouse models of aging and AD, where it has consistently demonstrated cognitive-enhancing effects, accompanied by improved mitochondrial function and decreased oxidative stress in the brain (Soumyanath et al., 2012; Gray et al., 2015, 2016, 2018a,b; Matthews et al., 2019, 2020; Zweig et al., 2021). However, we have not yet examined whether CAW also has an effect on neuroinflammation *in vivo*. Given the important role of neuroinflammation in the pathogenesis of AD and our previous research showing beneficial effects of CAW on pathways related to inflammation, an investigation into the effects of CAW on neuroinflammation *in vivo* is a logical next step in our evaluation of CAW as a potential herbal therapy for AD.

As an initial exploration of CAW’s effects on neuroinflammation, we analyzed the effect of CAW administered either in the drinking water or in the diet on Aβ pathology as well as neuroinflammatory markers in 5xFAD and wild-type (WT) mice using immunohistochemistry (IHC) and gene expression analyses. In addition, because we have observed a difference in the magnitude of the cognitive-enhancing effect when CAW was delivered in the drinking water (Matthews et al., 2019) versus in the diet (Matthews et al., 2020) to the mice evaluated in this study, we explored how plasma levels of CAW compounds varied with the mode of CAW administration, as a potential factor influencing the results.

## 2 Materials and methods

### 2.1 *Centella asiatica* water extract

Dried aerial parts of *Centella asiatica* herb were purchased from Oregon’s Wild Harvest (Redmond, OR, Lot #: 170300206) and authenticated by the supplier and at our lab, as described previously (Matthews et al., 2019). To prepare CAW, several batches of plant material were each boiled in distilled water under reflux for two hours in the ratio 160 g plant material to 2 L water. The resulting crude water extracts (CAW) were filtered, cooled, lyophilized, and stored as dried extracts at −20°C. Voucher samples of the original plant material and the extracts (CAW α-θ) are stored in our laboratory, and a voucher sample of the original plant material is kept at the Oregon State University Herbarium (OSC-V-258629). A representative batch of CAW (CAW-iota) was analyzed using a liquid chromatography-high resolution tandem mass spectrometry (LC-HRMS/MS) method described previously (Alcazar Magana et al., 2020). Quantification of individual caffeoylquinic acids (CQAs) and triterpenes (TTs) in CAW-iota based on co-analysis of reference standards has been published (Matthews et al., 2020; Speers et al., 2021). The full dataset of peaks detected by untargeted LC-HRMS/MS analysis of CAW iota is archived at Oregon State University.

### 2.2 Animals and CAW administration

All animal protocols were approved by the Portland VA Medical Center Institutional Animal Care and Use Committee (IACUC #: 3260-17) and were conducted in accordance with the NIH Guidelines for the Care and Use of Laboratory Animals. Male 5xFAD mice were bred with C57BL/6:SJL F1 females purchased from Jackson Laboratory (Bar Harbor, ME, United States). Presence or absence of transgenic APP in 5xFAD progeny and WT littermates, respectively, was confirmed via PCR using DNA from tail samples. A full description of the animals and their care and use has been published previously (Matthews et al., 2019, 2020). Brain tissue and plasma for the present study were collected from 5xFAD and WT animals that were treated with CAW (0 or 1,000 mg/kg/d) for five weeks, as described in our previously published studies (Matthews et al., 2019, 2020). The number of brain tissue samples that were used from each treatment group for each outcome measure is presented by in Supplementary Table S1.

In an initial study (Matthews et al., 2019), seven-to-eight-month-old male and female 5xFAD and WT mice received drinking water containing CAW (10 mg/mL) or plain drinking water (control) *ad libitum* (total of 8 treatment groups, *n* = 6–19 mice per group). CAW consumption was estimated at 1000 mg/kg/d based on mouse body weights, average daily decrease in supplied drinking water, and number of mice per cage. Mice were housed in a climate-controlled environment and provided with PicoLab Laboratory Rodent Diet 5LOD (LabDiet, St. Louis, MO, United States) and drinking water *ad libitum*.

In a subsequent study (Matthews et al., 2020), seven-to-eight-month-old male and female 5xFAD and WT mice received standardized AIN-93 M rodent diet (Dyets Inc., Bethlehem, PA, United States) as control or AIN-93 M diet containing CAW 1% w/w to approximate the 1,000 mg/kg/dose (based on mouse weights, food consumption, and number of mice per cage) as used in the previous CAW in drinking water study (Matthews et al., 2019) (8 treatment groups, *n* = 12–24 mice per group). The CAW diet was prepared by Dyets Inc. by mixing CAW with the AIN-93 M diet components until a homogenous distribution was achieved. After adding cold water (10%), the diet was run through a California Pellet Mill CL-3 lab pellet mill to create pellets, which were air dried at 27°C for 24 h. Finally, the diet was sterilized by gamma irradiation (5.0–20.0 kGy; Sterigenics, Oak Brook, IL, United States). Mice were housed in a climate-controlled environment and provided with the diets and drinking water *ad libitum*.

### 2.3 Immunohistochemistry

The right hemisphere of each mouse brain was fixed in 4% formaldehyde/PBS, transferred through a series of increasingly concentrated sucrose solutions (0 to 30%), and stored at −80°C. In preparation for IHC, samples were coronally sectioned at 40 microns using a freezing microtome. Each IHC run for Aβ included brain sections from all 5xFAD treatment groups, a positive control (brain section from human patient with AD), and a negative control (section of brain from WT mouse of same sex). Each IHC run for GFAP and GSL I included brain sections from all 5xFAD and WT treatment groups, and a positive control (brain section from human patient with AD). Sections were incubated in a quenching solution (30% methanol, 10% 10X TBS, and 0.3% H_2_O_2_ in distilled water) for endogenous catalase activity and blocked (10% horse serum, 10% 10X TBS, 2% BSA, 0.5% Triton X, and 0.01% sodium azide in distilled water) in 12-well plate Netwell inserts. Afterwards, sections were incubated overnight with 1:1000 pan Aβ antibody (44–136, Invitrogen, Carlsbad, CA, United States), 1:1000 glial fibrillary acidic protein (GFAP) antibody (PA1-10019, Invitrogen, Carlsbad, CA, United States), or 1:1000 *Griffonia simplicifolia* lectin I (GSL I) antibody (B-1105-2, Vector Laboratories, Burlingame, CA, United States). The following day, sections incubated with the pan Aβ antibody were labeled with 1:200 goat anti-rabbit pan Aβ secondary antibody (Vector laboratories, Burlingame, CA, United States). Sections incubated with the GFAP antibody were labeled with 1:200 biotinylated goat anti-rabbit GFAP secondary antibody (65–6,140, Invitrogen, Carlsbad, CA, United States). Staining for Aβ, GFAP, and GSL I was visualized with ABC kit (Vector Laboratories, Burlingame, CA) and DAB (Sigma Fast 3,3-Diaminobenzidine Tablet Set, D-4418, Sigma-Aldrich Corp., St. Louis, MO, United States) was used as a counterstain. After sections were mounted onto slides and dehydrated, slides were scanned with PrimeHisto XE (Pacific Image Electronics, Torrance, CA, United States). The analyst was blinded and three images per brain were selected for analysis. FIJI software (FIJI Is Just ImageJ, version 2.1.0) was used to quantify staining (Schindelin et al., 2012). Images were converted to 8-bit grayscale and the threshold was adjusted until only areas of intense staining were highlighted. The cortex or hippocampus was outlined with the polygon tool and the total area of each brain region was recorded. The number of individual areas staining higher than the threshold value are counted as “particles.” The FIJI analyze particles tool was used to calculate the percent area (i.e., the percentage of the total cortical or hippocampal area staining higher than the threshold value).

### 2.4 Gene expression

The left hemisphere of each mouse brain was sub-dissected into brain regions and frozen at −80°C. The deep grey tissue was used for gene expression analysis because the isolated cortical and hippocampal tissue from these animals had been exhausted in previous experiments. RNA was extracted from homogenized deep grey tissue with a QIAsymphony RNA kit (QIAGEN), using the RNA CT 400 v7 protocol. Reverse transcriptase was performed using a SuperScript^™^ VILO^™^ cDNA synthesis kit (Invitrogen). Relative gene expression was determined using custom Taqman^®^ Gene Expression Array Cards (Thermo Fisher Scientific) pre-loaded with the following commercially available Taqman primers: interleukin-6 (IL-6), tumor necrosis factor alpha (TNFα), interleukin-1 beta (IL-1β), triggering receptor expressed on myeloid cells 2 (TREM2), allograft inflammatory factor 1 (AIF1), C-X3-C motif chemokine receptor 1 (CX3CR1), C-X3-C motif chemokine ligand 1 (CX3CL1), cluster of differentiation 36 (CD36), complement C3a receptor 1 (C3AR1), receptor for advanced glycation endproducts (RAGE), CC motif chemokine receptor 6 (CCR6), CD3 epsilon subunit of t-cell receptor complex (CD3E), and glyceraldehyde-3-phosphate dehydrogenase (GAPDH). Quantitative PCR (qPCR) was performed using a QuantStudio^™^ 12 K Flex Real-time PCR System (Applied Biosystems) and a QuantStudio^™^ 7 K Flex Real-time PCR System (Applied Biosystems). Samples were run in triplicate and data was analyzed using the delta–delta Ct method normalized to GAPDH.

### 2.5 Plasma analysis

Blood was collected at sacrifice via cardiac puncture and centrifuged to collect plasma. Analytes measured in plasma were TTs: asiatic acid (AA), asiaticoside (AS), madecassic acid (MA), madecassoside (MS); monoCQAs: chlorogenic acid (CHLA), neochlorogenic acid (NA), cryptochlorogenic acid (CRYA); diCQAs: 1,3-dicaffeoylquinic acid (1,3-diCQA), 1,5-dicaffeoylquinic acid (1,5-diCQA), isochlorogenic acid A (IsoA), isochlorogenic acid B (IsoB), and isochlorogenic acid C (IsoC); and CQA metabolites: caffeic acid (CA), ferulic acid (FA), isoferulic acid (IFA), dihydrocaffeic acid (DHCA), dihydroferulic acid (DHFA), dihydroisoferulic acid (DHIFA) and 3-(3-hydroxyphenyl)propionic acid (HPP). Chrysin, and ^13^C_3_-ferulic acid (t*rans*-4-hydroxy-3-methoxycinnamic acid-^13^C_3_) were used as internal standards. CQAs were quantified as total monoCQAs (CHLA, NA, CRYA) and total diCQAs (1,3-diCQA, 1,5-diCQA, IsoA, IsoB, and IsoC), respectively; these compounds are able to isomerize by acyl migration (Alcázar Magaña et al., 2021), potentially altering their individual concentrations during sample preparation from the original values. The isobaric compounds DHIFA and DHFA co-elute and were analyzed together (reported as DHFA/DHIFA). CAS numbers and suppliers for reference compounds and internal standards are listed in Supplementary Table S2.

Plasma samples were prepared in duplicate for analysis by HPLC–MS/MS using methods previously described (Cheng et al., 2003; Wright et al., 2022). Plasma samples (50 μL) and an ascorbic acid solution (1%; 10 μL) were combined with *A. aerogenes* sulfatase (2.5 μL; 0.025–0.05 units; Sigma-Aldrich) and *E. coli glucuronidase* (2.5 μL; 12.5–125 units; Sigma-Aldrich) dissolved in 50% aqueous glycerol. The mixture was incubated at 37°C for 20 min to release the analytes from potential sulfate and β-D-glucuronic acid conjugates. Protein precipitation was then performed by adding a crash solution (200 μL) made up of acetonitrile:methanol (3:1) containing internal standards chrysin (25 ng/mL) and ^13^C_3_-ferulic acid (62.5 ng/mL), holding for 30 min at 4°C, and then centrifuging for 5 min at 10,000 X g. The supernatant was filtered through a 0.22 μm Ultrafree MC-GV spin filter (Millipore Sigma) at 10,000 X g for 5 min at 4°C before being transferred into two separate HPLC vials for TT or CQA analysis. To optimize peak shape in the HPLC–MS/MS for TT analysis, the concentration of organic solvent to water in each sample was adjusted to a 60:40 ratio; for CQA and CQA metabolite analysis, the concentration of organic solvent to 1% aqueous formic acid in each sample was adjusted to a 90:10 ratio.

For the detection of TT glycosides and their associated aglycones, a modification of a method previously described (Nair et al., 2012) was used. HPLC-MS/MS was performed on an Applied Biosystems Q-Trap 4000 LC-MS instrument (Framingham, MA, United States). Chromatographic separation was achieved using a Poroshell 120 EC18 column (3 mm id x 50 mm; 2.7 μ), Poroshell ultra high-performance liquid chromatography (UHPLC) guard column, and a methanol:ammonium acetate pH gradient (Santa Clara, CA, United States). The injection volume was 20 μL. Gradient elution was performed using a mobile phase of solvent A (water containing 10 mM ammonium acetate and 0.02% ammonium hydroxide; pH 8.5) and solvent B (methanol). The flow rate was 0.42 mL/min. The chromatographic method duration was 9 min, and the gradient design was as follows: an initial 2 min increase from 40–60% B, followed by 60–95% B from 2–3.5 min, hold at 95% B from 3.5–6 min, return to 40% B by 6.1 min, and re-equilibrate at 40% B from 6.1–9 min. TTs were detected as their ammonium adducts with positive ion mode electrospray ionization. Quantification was performed using chrysin as internal standard.

For the detection of CQAs and their associated metabolites, HPLC-MS/MS was performed on an Applied Biosystems 5500 QTRAP HPLC-MS instrument (Framingham, MA, United States). Chromatographic separation was achieved using a C8 reversed-phase column (Agilent Zorbax Eclipse plus C8 Rapid resolution 4.6x150mm 3.5 μ; Santa Clara, CA, United States), Agilent Zorbax Eclipse plus C8 Rapid resolution guard column (4.6×12.5 mm 5 μ; Santa Clara, CA, United States) and acidified acetonitrile:water gradient. The injection volume was 5 μL. Gradient elution was performed using a mobile phase of solvent A (water containing 0.05% v/v acetic acid) and solvent B (acetonitrile with 0.05% v/v acetic acid). The flow rate was 0.8 mL/min. The chromatographic method had a duration of 21 min, and the gradient design was as follows: an initial 0.1 min at 10% B, an increase to 25% B by 4.5 min, 25–40% B from 4.5–10 min, 40 to 95% B from 10–11 min, hold at 95% B from 11–16 min, return to 10% B by 16.2 min, and re-equilibrate at 10% from 16.2–21 min. CQAs and their metabolites were detected using negative ion mode electrospray ionization. Quantification was performed using ^13^C_3_-ferulic acid as internal standard.

The MS/MS transitions used for detection of the analytes and internal standards are listed in Supplementary Table S3. Analyte peaks in each sample were identified using Analyst software from Sciex Technologies (version 1.7.1). Peak area, peak height, and retention time were recorded for each analyte and internal standard. When an analyte had multiple MS/MS transitions, the transition with the greatest peak area, smoothest peak shape, and minimal baseline noise was used for analysis. Peak area ratios of analyte to internal standard were calculated for each sample. Analytes were quantified by reference to calibration curves of peak area ratio of analyte to internal standard prepared using commercial reference standards spiked into untreated mouse plasma.

### 2.6 Statistical analysis

Prior to formal analysis, outcome measures were examined univariately to determine distribution and possible outliers that might suggest warranted transformation. Bivariate plots of the analytes by grouping variables were also constructed for the same purpose. Prior to final model fitting, a Box-Cox transformation was applied to the response variable to better adhere to model assumptions (normality, heterogenous error variance, etc.). A linear regression model with a three-way interaction between treatment (CAW and control), treatment medium (diet and drinking water), and genotype (5xFAD and WT) was then fit to the transformed response separately for each sex. *A priori* contrasts between treatment and genotype were performed on this transformed scale within each treatment medium and a Holm-Bonferroni adjustment was applied to contrast *p*-values to control for the family-wise error rate.

## 3 Results

### 3.1 CAW in drinking water reduces cortical Aβ plaque burden in 5xFAD female mice

**Figure 1:**
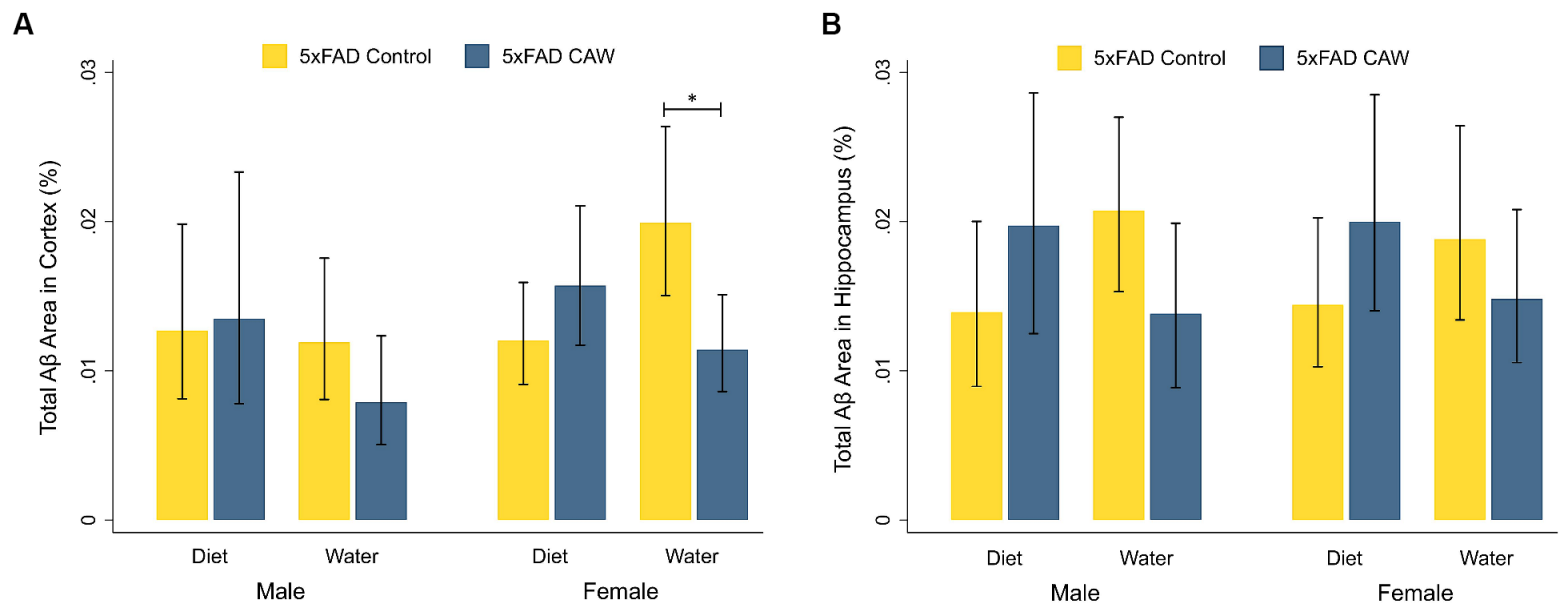
CAW (1,000 mg/kg/d for five weeks) only reduces Aβ plaque burden in the cortex of 5xFAD female mice when delivered in the drinking water. Total Aβ area (%) in the **(A)** cortex and **(B)** hippocampus of male and female CAW-and control-treated 5xFAD mice. *n* = 6–12 per treatment group, *p < 0.05.

We analyzed the Aβ plaque burden in the cortex and hippocampus of 5xFAD mice that had received CAW in the drinking water or in the diet (0 or 1,000 mg/kg/d) for five weeks total (Figure 1). For male 5xFAD mice, there was not a significant difference in cortical or hippocampal Aβ staining between CAW-treated and control mice for either mode of administration. Similarly, for 5xFAD female mice administered CAW in the diet, cortical and hippocampal Aβ staining were not significantly different between CAW-treated and control mice. CAW did, however, decrease cortical Aβ staining in 5xFAD female mice administered CAW in the drinking water compared to control (*p* = 0.02). No significant differences in cortical or hippocampal Aβ staining were seen when comparing animals administered CAW in the diet versus in the drinking water for either sex.

### 3.2 CAW does not alter cortical or hippocampal staining of the astrocytic marker GFAP in 5xFAD mice

**Figure 2:**
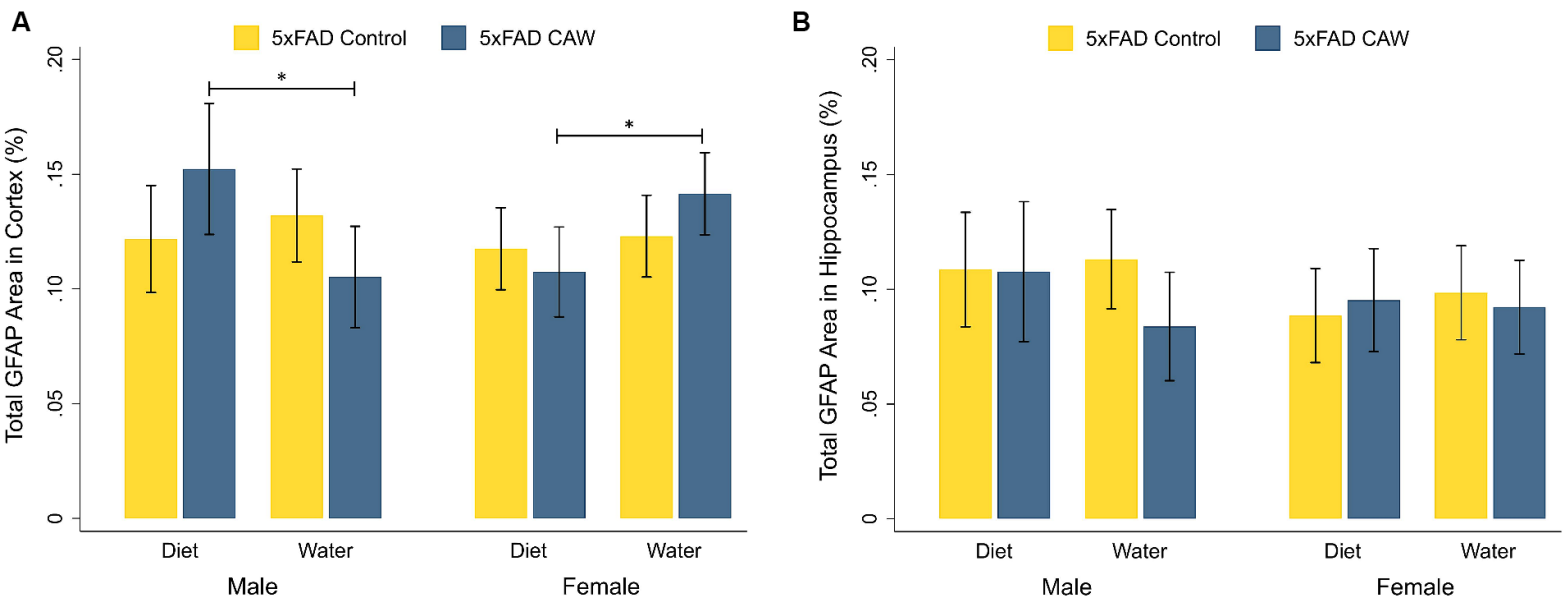
CAW (1,000 mg/kg/d for 5 weeks) does not alter GFAP in the cortex or hippocampus of 5xFAD mice when administered in the diet or the drinking water. Total GFAP area (%) in the **(A)** cortex and **(B)** hippocampus of male and female CAW-and control-treated 5xFAD mice. *n* = 6–12 per treatment group, *p < 0.05.

As expected, GFAP staining in the cortex and hippocampus was significantly higher in control 5xFAD mice compared to their WT littermates, for both sexes (Supplementary Figure S1). CAW did not significantly alter cortical or hippocampal GFAP staining in CAW-treated 5xFAD mice compared to control 5xFAD mice, for either sex or mode of CAW administration (Figure 2). In males, cortical GFAP staining was significantly higher in 5xFAD mice administered CAW in the diet versus in the drinking water (*p* = 0.04). The opposite effect was observed in female mice; cortical GFAP staining was significantly higher in 5xFAD female mice administered CAW in the drinking water versus in the diet (*p* = 0.04). There were no significant differences in hippocampal GFAP staining between 5xFAD mice administered CAW in the diet versus in the drinking water for either sex.

### 3.3 CAW does not alter cortical or hippocampal staining of the microglial marker GSL I in 5xFAD mice

**Figure 3:**
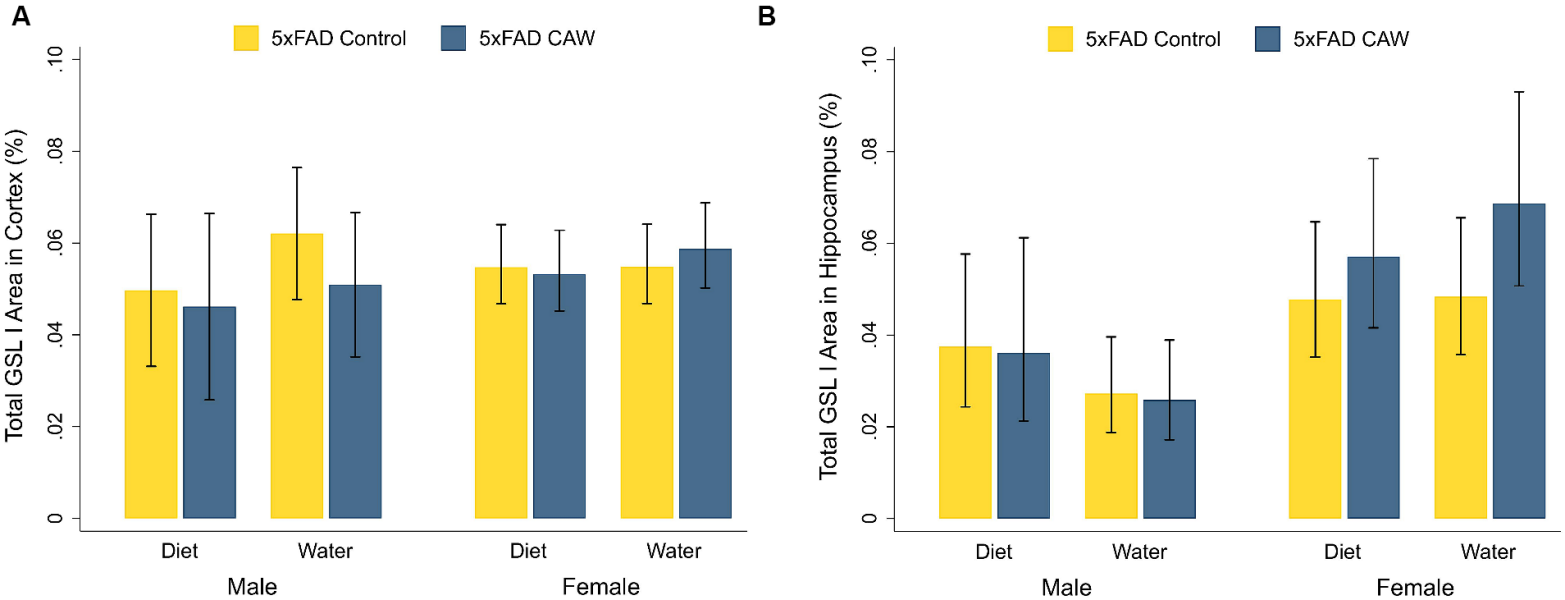
CAW (1,000 mg/kg/d for 5 weeks) does not alter GSL I in the cortex or hippocampus of 5xFAD mice when delivered in the diet or in the drinking water. Total GSL I area (%) in the **(A)** cortex and **(B)** hippocampus of male and female CAW-and control-treated 5xFAD mice. *n* = 6–12 per treatment group.

Cortical and hippocampal staining for GSL I was significantly higher in control 5xFAD mice compared to their WT littermates, for both sexes (Supplementary Figure S2). CAW did not significantly alter cortical or hippocampal staining for GSL I in CAW-treated 5xFAD mice compared to control 5xFAD mice, for both sexes and both modes of CAW administration (Figure 3). In addition, there were no significant differences in cortical or hippocampal GSL I staining between 5xFAD mice treated with CAW in the diet versus in the drinking water for either sex.

### 3.4 CAW in drinking water alters deep grey C3AR1 gene expression in 5xFAD female mice, while CAW in diet alters deep grey IL-1β gene expression in 5xFAD male mice

Expression of TNFα, IL-1β, TREM2, AIF1, C3AR1, and CXC3R1 were all significantly higher in 5xFAD mice compared to their WT littermates for both sexes (Supplementary Table S4). Gene expression of CD3E was significantly higher only in 5xFAD males used in the diet experiments compared to WT mice. A similar but non-significant increase in CD3E expression was observed in 5xFAD males used in the water experiments and the 5xFAD females in both the diet and water experiments. There were no significant differences observed in the gene expression of IL-6, CX3CL1, CD36, RAGE, or CCR6 between 5xFAD and WT control mice for either sex (Supplementary Table S4).

CAW delivered in the drinking water significantly increased C3AR1 expression in the deep grey of 5xFAD female mice compared to 5xFAD control mice, while CAW delivered in the diet significantly decreased IL-1β expression in the deep grey of 5xFAD male mice compared to 5xFAD control mice (Table 1). CAW did not alter C3AR1 or IL-1β expression in the deep grey for any other treatment group and did not alter the expression of other inflammatory mediators (IL-6, TNFα, TREM2, AIF1, CX3CR1, CX3CL1, CD36, RAGE, CCR6, or CD3E) in the deep grey for either sex or mode of administration (Table 1).

**Table 1:** Gene expression of inflammatory markers in the deep grey matter of CAW-treated 5xFAD mice.

		Female		Male	
Cytokine	Treatment group	Fold change	SEM	Fold change	SEM
IL-6	CAW water	1.11	0.21	0.54	0.05
	CAW diet	0.81	0.16	1.16	0.29
TNFα	CAW water	1.08	0.16	0.74	0.19
	CAW diet	1.04	0.20	0.73	0.14
IL-1β	CAW water	0.90	0.17	1.20	0.18
	CAW diet	2.51	1.29	0.46*	0.11
TREM2	CAW water	1.69	0.17	0.62	0.09
	CAW diet	1.02	0.21	0.53	0.08
AIF1	CAW water	1.35	0.14	0.91	0.11
	CAW diet	1.02	0.18	0.69	0.09
CX3CR1	CAW water	1.59	0.17	0.92	0.13
	CAW diet	1.14	0.21	0.85	0.20
CX3CL1	CAW water	1.12	0.12	1.25	0.30
	CAW diet	1.30	0.24	0.97	0.12
CD36	CAW water	1.24	0.14	1.23	0.25
	CAW diet	1.42	0.25	1.14	0.21
C3AR1	CAW water	1.56*	0.15	0.91	0.16
	CAW diet	1.04	0.13	0.66	0.10
RAGE	CAW water	1.53	0.16	1.05	0.15
	CAW diet	0.83	0.16	1.36	0.38
CCR6	CAW water	1.51	0.24	1.20	0.16
	CAW diet	0.74	0.31	1.05	0.19
CD3E	CAW water	1.31	0.17	0.85	0.15
	CAW diet	0.85	0.17	0.82	0.17

*n* = 6–12 per treatment group, *p < 0.05. Values represent the fold change for CAW-treated 5xFAD animals compared to control 5xFAD mice for a given mode of CAW administration.

### 3.5 CAW administered in the drinking water versus in the diet results in higher plasma concentrations of several CAW compounds

#### 3.5.1 TTs

**Figure 4:**
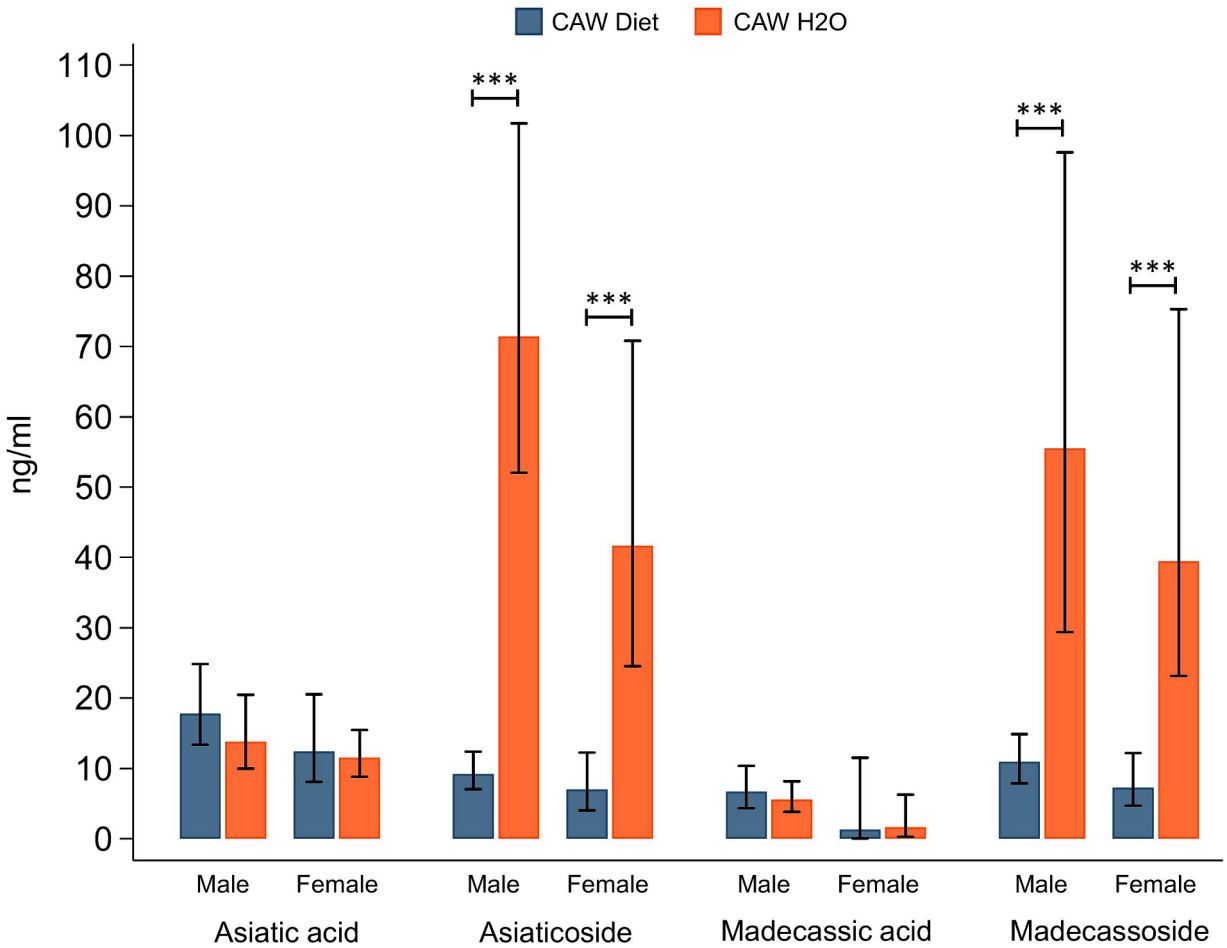
Plasma levels of triterpene glycosides were significantly higher in mice receiving CAW (1,000/mg/kg/d) in the drinking water versus in the diet.

Plasma concentrations of CAW TTs in male and female 5xFAD animals administered CAW are shown in Figure 4. There were no significant differences in plasma concentrations for either aglycone (asiatic acid, madecassic acid) when comparing 5xFAD mice administered CAW in the diet versus in the drinking water. Interestingly however, plasma concentrations of TT glycosides (asiaticoside, madecassoside) were significantly higher in 5xFAD mice administered CAW in the drinking water compared to those administered CAW in the diet; this finding was observed for both 5xFAD males (asiaticoside: *p* < 0.001, madecassoside: *p* < 0.001) and 5xFAD females (asiaticoside: *p* < 0.001, madecassoside: *p* < 0.001). There were no significant sex-related differences in TT concentrations, for either mode of administration (Figure 4).

#### 3.5.2 CQAs

**Figure 5:**
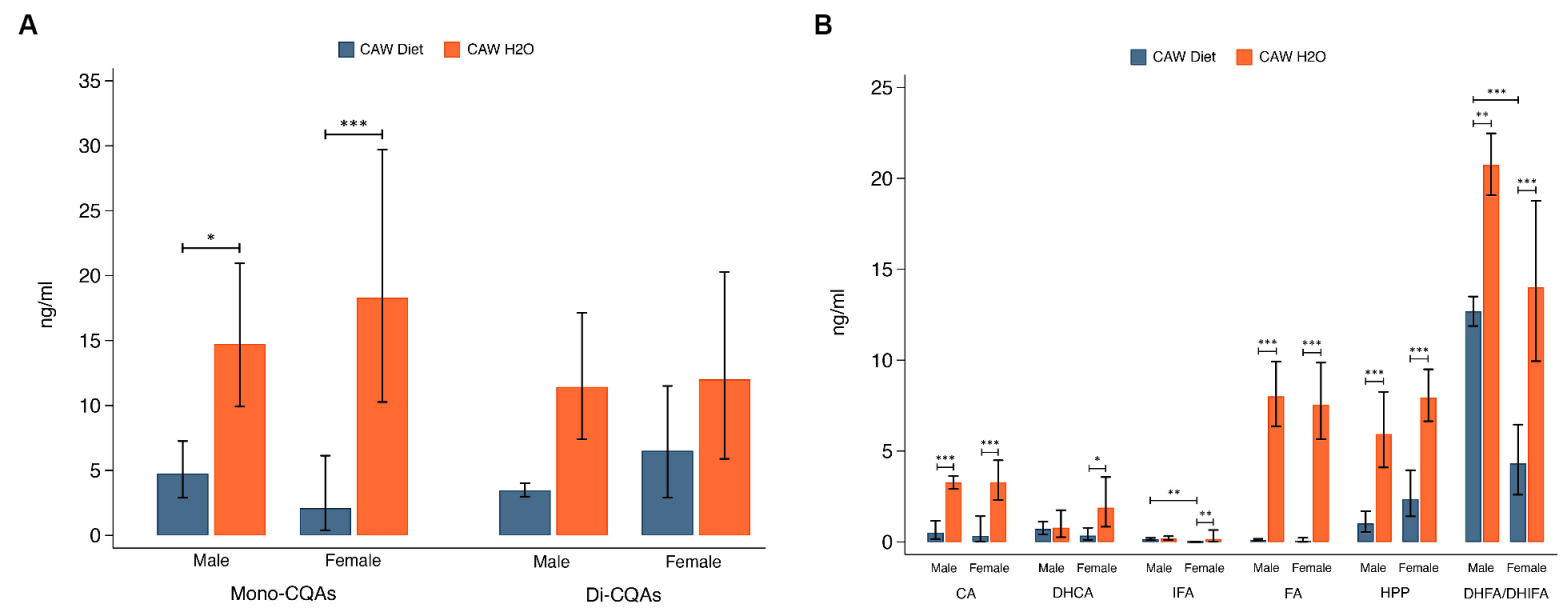
Plasma concentrations of caffeoylquinic acids (CQAs) and CQA metabolites were significantly higher in 5xFAD mice administered CAW (1,000 mg/kg/d) in the drinking water versus in the diet. **(A)** Plasma concentrations of monoCQAs and diCQAs in CAW-treated male and female 5xFAD mice. **(B)** Plasma concentrations of CQA metabolites in CAW-treated male and female 5xFAD mice. *n* per treatment group = 7–12, *p < 0.05, **p < 0.01, ***p < 0.001.

Plasma concentrations of total mono-CQAs (CHLA, NA, CRYA) and di-CQAs (1,3-diCQA, 1,5-diCQA, IsoA, IsoB, IsoC) are presented in Figure 5A. Plasma concentrations of mono-CQAs were significantly higher in 5xFAD mice administered CAW in the drinking water versus in the diet for both sexes (males: *p* < 0.05, females: *p* < 0.001). There was not a significant difference in plasma concentrations of di-CQAs between the two modes of CAW administration for either sex. Figure 5B shows the plasma concentrations of six CQA metabolites. Plasma concentrations of four CQA metabolites (CA, FA, HPP, DHFA/DHIFA) were significantly higher in 5xFAD mice administered CAW in the drinking water versus in the diet for both sexes (CA, FA, HPP: *p* < 0.001 for both sexes; DHFA/DHIFA: *p* < 0.01 for males, *p* < 0.001 for females). In female 5xFAD mice, plasma concentrations of DHCA (*p* < 0.05) and IFA (*p* < 0.01) were also significantly higher in those animals administered CAW in the drinking water versus in the diet, while no significant difference was observed in male 5xFAD mice. There were two significant sex-related differences observed. In animals administered CAW in the diet, plasma levels of IFA and DHFA/DHIFA were significantly higher in males versus females (Figure 5). There were no sex-related differences in plasma compound levels among animals administered CAW in the drinking water.

## 4 Discussion

Our lab has previously reported that CAW improves cognition in mouse models of aging and AD, and that the cognitive-enhancing effects of CAW are accompanied by reduced oxidative stress and improved mitochondrial function (Soumyanath et al., 2012; Gray et al., 2015, 2016, 2018a,b; Matthews et al., 2019, 2020; Zweig et al., 2021). Here, we sought to further elucidate the mechanisms underlying the cognitive benefits of CAW by investigating whether CAW impacts neuroinflammation in the 5xFAD mouse model of Aβ accumulation (Oakley et al., 2006). We began by investigating whether CAW altered the Aβ plaque burden in the cortex and hippocampus of 5xFAD mice. We then assessed the effect of CAW on protein markers of reactive astrocytes (GFAP) and microglia (GSL I) in the cortex and hippocampus and gene expression of several inflammatory mediators (IL-6, TNFα, IL-1β, TREM2, AIF1, CX3CR1, CX3CL1, CD36, C3AR1, RAGE, CCR6, and CD3E) in the deep grey matter of 5xFAD animals. The brain tissue samples included in our analysis were obtained from two separate experiments where CAW had been administered either in the drinking water (Matthews et al., 2019) or in the diet (Matthews et al., 2020) at a calculated dose of 1,000 mg/kg/day for five weeks. We therefore also explored whether the mode of CAW administration (diet versus drinking water) affected our outcomes and whether plasma levels of CAW compounds were influenced by how CAW was administered.

A notable finding in our study was that, of the 12 CAW-derived compounds we measured in the plasma, seven were present in significantly higher concentrations when CAW was administered in the drinking water versus in the diet for both male and female 5xFAD mice (Figures 4, 5). For several of these comparisons (e.g., asiaticoside, madecassoside), the difference in plasma levels between the two modes of administration was dramatic, with concentrations four to seven times higher in the water administration cohorts. There were two significant sex-related differences in 5xFAD animals administered CAW in the diet (IFA and DHFA/DHIFA were higher in males versus females), but otherwise no sex-related differences were observed. The significant effect of mode of administration on plasma levels of CAW compounds was unexpected, though this may explain previous results in these 5xFAD mice where CAW delivered in the diet produced a muted behavioral response (Matthews et al., 2020) compared to the response when CAW had been administered in the drinking water (Matthews et al., 2019). Absorption of active compounds from CAW may be impaired when delivered in the diet due to variations in gut transit time, gut microbial transformation, or reduced release of CAW compounds from the diet. These findings will inform the design of our future preclinical and clinical research on CAW.

CAW administered in the drinking water significantly decreased Aβ plaque burden in the cortex of female 5xFAD mice compared to control 5xFAD mice but did not impact Aβ plaque burden in the hippocampus or in male mice, nor was there any effect on Aβ plaque burden in either sex when CAW was administered in the diet. The difference in the effect of CAW on Aβ in the cortex of 5xFAD female mice between the two modes of administration was accompanied by differences in plasma levels of the active compounds. However, the plasma levels were similar between male and female 5xFAD mice administered CAW in the drinking water. Therefore, compound plasma levels alone do not appear to account for differences seen between the treatment groups.

In our previous experiments, the effects of CAW on Aβ plaque burden have been inconsistent. A study in a cohort of Tg2576 mice found no effect of CAW on brain levels of soluble or insoluble Aβ after two weeks’ administration in the drinking water (Soumyanath et al., 2012). In 5xFAD mice administered CAW in the drinking water for five weeks, CAW had no effect on Aβ plaque burden in the cortex or hippocampus for either male or female mice (Matthews et al., 2019). The mice used in this previous experiment are the same mice used in the present study where a significant effect on Aβ plaque burden was seen in the cortex of 5xFAD female mice administered CAW in the drinking water. However, in the previous study, only 5–7 animals were used per treatment group, whereas our analyses used 7–12 animals per group (Supplementary Table S1), which may have provided the statistical power required to see the effects of CAW on Aβ plaque burden. A study in which only female 5xFAD mice were administered CAW in the drinking water for five weeks found that CAW decreased Aβ plaque burden in the hippocampus, but not the cortex (Gray et al., 2018b). However, in a cohort of four-month-old 5xFAD mice administered CAW in the drinking water for three months, CAW significantly decreased Aβ plaque burden in the cortex of both male and female 5xFAD mice compared to untreated controls; CAW also significantly decreased Aβ plaque burden in the hippocampus of these female 5xFAD mice, but not the males (Zweig et al., 2021). Collectively, this evidence suggests CAW administered in the drinking water could impact Aβ plaque burden, but that this effect may vary by sex, brain region, age of animals, and duration of treatment.

As expected, based on previous reports in the literature, neuroinflammatory markers were significantly higher in 5xFAD mice compared to WT mice. IHC staining for reactive astrocytes (GFAP) and microglia (GSL I) was significantly higher in control 5xFAD mice compared to their WT littermates; this finding was observed for both male and female mice and in both the cortex and hippocampus. These results are in line with previous research showing that 5xFAD mice have significantly higher levels of reactive astrogliosis and microglia in the cortex and hippocampus compared to WT mice (Forner et al., 2021). In the deep grey, gene expression of TNFα, IL-1β, TREM2, AIF1, C3AR1, and CXC3R1 was significantly higher for control 5xFAD mice compared to their WT littermates, though there was no difference in gene expression of IL-6, CX3CL1, CD36, RAGE, or CCR6. The cytokine findings are consistent with a recent characterization of the 5xFAD mouse model published after our experiments were completed that revealed elevated levels of TNFα and IL-1β, but not IL-6, in hemibrain samples from 5xFAD mice (Oblak et al., 2021).

Contrary to our hypotheses however, we found little evidence that CAW has an effect on any of the selected markers of neuroinflammation. When comparing CAW-treated and control 5xFAD animals, no significant differences were observed for cortical or hippocampal staining of GFAP or GSL I for either sex or mode of administration; similarly, no significant differences were observed in the gene expression of IL-6, TNFα, TREM2, AIF1, CX3CR1, CX3CL1, CD36, RAGE, CCR6, or CD3E in the deep grey for either sex or mode of administration. There was a significant decrease in the expression of the pro-inflammatory cytokine IL-1β in 5xFAD male mice administered CAW in the diet and, interestingly, an increase in the complement receptor C3AR1 gene expression in the deep grey of 5xFAD female mice administered CAW in the water. Future experiments are needed to elucidate the implications of these changes in individual genes.

The overall lack of effect for CAW on markers of neuroinflammation was consistent across both sexes and both modes of CAW administration. These findings were somewhat unexpected based on the existing literature, where various extracts of *Centella asiatica* have demonstrated anti-inflammatory activity across a wide range of *in vitro* (Abdullah et al., 2012; Hao et al., 2017; Naidoo et al., 2017; Cao et al., 2018; Moolsap et al., 2020) and *in vivo* models (Guo et al., 2004; Sharma et al., 2014; Masola et al., 2018; Hafiz et al., 2020), though only two of these studies were conducted with a water extract of *Centella asiatica*. *In vitro*, a water extract of *Centella asiatica* inhibited COX-1 and COX-2 enzymes and decreased inflammatory prostaglandins in a human fibroblast model of TPA-induced inflammation (Abdullah et al., 2012), while in a mouse model of gastric ulcers, a water extract of *Centella asiatica* inhibited nitric oxide synthesis (Guo et al., 2004). It is notable that, unlike the present investigation, none of these earlier studies examined inflammatory end points in the brain. There may be a difference between the peripheral and central anti-inflammatory effects of CAW based on the brain bioavailability of (as yet uncharacterized) anti-inflammatory compounds in CAW.

Furthermore, our findings were surprising given the results of our earlier experiments on the mechanisms underlying the cognitive effects of CAW. We have previously demonstrated that the cognitive benefits of CAW are accompanied by reduced oxidative stress and improved mitochondrial function in the brain in mouse models of AD (Soumyanath et al., 2012; Gray et al., 2015, 2018b; Matthews et al., 2019; Zweig et al., 2021), two pathways inextricably linked with the neuroinflammatory response. Evidence suggests that neuroinflammation promotes and worsens the Aβ and tau pathologies found in AD (Kinney et al., 2018). Aβ and tau pathologies trigger a dysfunctional, proinflammatory state in mitochondria whereby energy production shifts from oxidative phosphorylation to glycolysis (Onyango et al., 2021). Mitochondria, which are a primary source of reactive oxygen species (ROS) within cells, increase their production of ROS when their function is impaired (Onyango et al., 2021). The subsequent rise in oxidative stress further stimulates the inflammatory response, creating a relationship where neuroinflammation is thought to be both a cause and effect of oxidative stress in AD (Hampel et al., 2020). Given the intertwined relationship between mitochondrial dysfunction, oxidative stress, and neuroinflammation in AD, it was surprising that despite previous observations of an ameliorating effect of CAW on oxidative stress and mitochondrial dysfunction, we did not find any evidence of an effect on neuroinflammation.

The lack of an observed anti-inflammatory effect of CAW in the 5xFAD mouse brain, despite the observation of robust cognitive effects, could be the result of several factors. First, it’s possible that the beneficial effects of CAW on cognition are not driven by an anti-inflammatory mechanism, though there is evidence that CAW activates the nuclear factor erythroid 2-related factor 2 (NRF2) mediated antioxidant response pathway in 5xFAD animals (Gray et al., 2018b; Matthews et al., 2019; Zweig et al., 2021). In a metabolomics study of 5xFAD mice administered CAW in the drinking water, we found three pathways (nicotinate and nicotinamide metabolism, purine metabolism, glycerophospholipid metabolism) that were significantly altered by CAW in 5xFAD males and females (Speers et al., 2021). In the case of nicotinate and nicotinamide metabolism, for example, we found that CAW upregulated cortical levels of NAD+ in 5xFAD males and females. This finding was notable because NAD+ and its precursors have previously demonstrated cognitive-enhancing effects in various mouse models of AD (Gong et al., 2013; Liu et al., 2013; Wang et al., 2016; Xing et al., 2019). Similarly, in the glycerophospholipid metabolism pathway, CAW upregulated cortical levels of sn-glycero-3-phosphocholine in 5xFAD males and females. Also known as glycerophosphocholine, this compound is an acetylcholine precursor that has demonstrated cognitive-enhancing effects *in vitro* (Catanesi et al., 2020), *in vivo* (Lee et al., 2017), and in early human trials (Carotenuto et al., 2017). These findings were exploratory in nature and have not yet been confirmed but do offer preliminary evidence for other mechanisms by which CAW may exert its cognitive-enhancing effects.

Another reason for the lack of an observed anti-inflammatory effect could be the result of the molecular markers we chose to study. GFAP is a commonly used marker for reactive astrogliosis, a process by which astrocytes, the most common cells in the central nervous system, undergo morphological changes in response to various stimuli (Pekny and Pekna, 2004; Escartin et al., 2021). As part of this process, astrocytes increase their expression of GFAP, an intermediate filament protein (Pekny and Pekna, 2004). Reactive astrocytes are not a homogenous population, however, and can fluctuate between states depending on the context (Escartin et al., 2021). A recent study examining the 5xFAD model, for example, characterized six unique clusters of astrocytes, several of which demonstrate higher levels of GFAP expression (Habib et al., 2020). Notably, a unique population of “disease-associated astrocytes” was identified that appear early in AD and increase with disease progression, and which are associated with the upregulation of specific genes involved in amyloid accumulation, metabolism, and clearance (Habib et al., 2020). Therefore, measuring GFAP alone is insufficient for determining whether CAW impacts AD-related reactive astrogliosis, and future studies should include additional markers (e.g., genes whose upregulation is linked to “disease-associated astrocytes” such as Serpina3n, Ctsb, Apoe, and Clu) to investigate whether CAW may be altering specific subpopulations of astrocytes.

A similar case could be made for our choice of a marker for reactive microglia, GSL I. Like GFAP with astrocytes, staining for GSL I can identify microglia in a reactive state, but does not provide information regarding the functional nature of that state. While reactive microglia have been commonly classified into a pro-inflammatory M1 phenotype and a neuroprotective M2 phenotype (Hampel et al., 2020), newer technologies have demonstrated that microglia in a reactive state do not polarize to either of these phenotypes and can often co-express markers associated with both phenotypes (Paolicelli et al., 2022). Several microglial transcriptional signatures have been identified in AD, including disease-associated microglia (DAM), microglia neurodegenerative phenotype (MGnD), and activated response microglia (ARM), all associated with specific sets of upregulated genes (Paolicelli et al., 2022). Future studies of CAW in 5xFAD mice could include analysis of these genes (e.g., Apoe, Trem2, Clec7a, Spp1, Lpl, Cst7, Ctsd) to investigate how CAW impacts various disease-associated reactive states for microglia.

The only significant effects observed on gene expression levels of inflammatory mediators in the deep grey was an increase in C3AR1 expression in 5xFAD females administered CAW in the drinking water and a decrease in IL-1β in 5xFAD males administered CAW in the diet. C3AR1 is a receptor in the complement system that is expressed in the brain by microglia, astrocytes, and neurons (El Gaamouch et al., 2020). Data for C3AR1’s role in AD is limited, but a study in PS19 mice found that inactivation of C3AR1 was associated with *decreased* neuroinflammation and tau pathology (Litvinchuk et al., 2018). IL-1β is a pro-inflammatory cytokine that drives neuroinflammation in AD but also has a protective effect on the CNS depending on the context (Boraschi et al., 2023). These findings could be relevant to CAW’s underlying mechanisms but were specific to one sex and one mode of administration in each case, making it difficult to draw any strong conclusions. To further investigate the effect of CAW on inflammatory mediators, future studies could examine the effect of CAW on inflammatory mediators (including anti-inflammatory cytokines) in brain regions where a biological effect of CAW has previously been demonstrated (i.e., cortex and hippocampus). Unfixed tissue samples from those brain regions had been used in previous studies and were not available for inclusion in this study’s gene expression analysis.

Despite these interesting findings, our study is not without limitations. As discussed, our choice of neuroinflammatory IHC markers was insufficient and future studies should incorporate markers that have been associated with disease-specific states of astrocytes and microglia. The lack of additional cortical and hippocampal tissue for our studies of inflammatory mediators was also a limitation in that it prevented us from directly comparing brain regions where we had performed IHC. As shown in Supplementary Table S1, we did not have a consistent number of animals in each group. The 5xFAD CAW Diet group (*n* = 6), in particular, was limited due to previous storage issues that prevented the use of several brains. It’s also notable that the animals used in this study were treated with CAW for five weeks. As mentioned in the case of Aβ plaque burden, a longer duration of CAW treatment produced more pronounced effects (Zweig et al., 2021), suggesting that the same could potentially be true for some of the markers chosen in this study. Finally, it may be helpful to quantify individual animal consumption of food and water in future studies as a potential factor impacting the difference in plasma levels of CAW compounds observed between the two modes of administration.

In summary, we found no evidence of an anti-inflammatory effect for CAW in the brains of 5xFAD mice. While it’s possible CAW enhances cognition without having an effect on neuroinflammation, further research is needed to investigate CAW’s potential anti-inflammatory effects. Future studies should incorporate additional molecular markers, morphological analysis, and cell function assessments to better describe the effect of CAW on astrocytes and microglia in the setting of AD. Based on the results of our plasma analysis, future studies should prioritize administering CAW in the drinking water rather than the diet as plasma levels of CAW compounds were significantly higher using this mode of administration.

## Data availability statement

The original contributions presented in the study are publicly available. This data can be found at: https://figshare.com/projects/Alzheimer_s_Disease_Supplement_Project/178668.

## Ethics statement

The animal study was approved by Portland VA Medical Center Institutional Animal Care and Use Committee. The study was conducted in accordance with the local legislation and institutional requirements.

## Author contributions

ABS: Data curation, Investigation, Methodology, Visualization, Writing – original draft, Writing – review & editing. KW: Investigation, Methodology, Writing – original draft, Writing – review & editing. MB: Investigation, Writing – review & editing. NK: Investigation, Writing – original draft, Writing – review & editing. DM: Investigation, Writing – review & editing. MC: Investigation, Writing – review & editing. CH: Investigation, Supervision, Writing – review & editing. SK: Formal analysis, Writing – review & editing. TN: Formal analysis, Writing – review & editing. JQ: Resources, Supervision, Writing – review & editing. AmS: Conceptualization, Funding acquisition, Methodology, Resources, Supervision, Writing – review & editing. NG: Conceptualization, Funding acquisition, Investigation, Methodology, Project administration, Resources, Supervision, Writing – review & editing.
