# Supplementary material for: Mode of administration influences plasma levels of active Centella asiatica compounds in 5xFAD mice while markers of neuroinflammation remain unaltered
Source: Front Neurosci. 2024 Mar 25;18:1277626. doi: 10.3389/fnins.2024.1277626 (PMC10999680; doi:10.3389/fnins.2024.1277626)
Supplement: Supplementary file 1 [file Data_Sheet_1.pdf]

**Supplementary Materials**

# Mode of administration influences plasma levels of active *Centella asiatica* compounds in 5xFAD mice while markers of neuroinflammation remain unaltered

**Alex B. Speers[1,2*], Kirsten M. Wright[1,2], Mikah S. Brandes[1,2], Nareg Kedjejian[1,2], Donald G. Matthew[2], Maya Caruso[2], Christopher J. Harris[2], Seiji Koike[1,3], Thuan Nguyen[1,3], Joseph F. Quinn[1,2,4], Amala Soumyanath[1,2], and Nora E. Gray[1,2]**

[1] BENFRA Botanical Dietary Supplements Research Center, Oregon Health & Science University, Portland, OR, USA
[2] Department of Neurology, Oregon Health & Science University, Portland, OR, USA
[3] School of Public Health, Oregon Health & Science University-Portland State University, Portland, OR, USA.
[4] Parkinson's Disease Research Education and Clinical Care Center, Veterans' Administration Portland Health Care System, Portland, OR, USA

*** Correspondence:**
Alex Speers
speers@ohsu.edu

Supplementary Figures 1 and 2 show the difference in GFAP and GSL I staining in the cortex and hippocampus between 5xFAD control and WT control mice. The brain tissue samples used in these analyses were from two previous mouse experiments, referred to as Study 1 and Study 2 in the figures. In Study 1 (Matthews et al, 2020), 5xFAD and WT control mice were fed AIN-93M diet (Dyets Inc., Bethlehem, PA, USA). In Study 2 (Matthews et al, 2019), 5xFAD and WT control mice were fed PicoLab Laboratory Rodent Diet 5L0D (LabDiet, St. Louis, MO, USA). The drinking water was the same in both studies.

**Supplementary Figure 1**. 5xFAD mice show significantly higher levels of GFAP staining in the cortex and hippocampus versus WT mice. Total GFAP area (%) in the (A) cortex and (B) hippocampus of male and female WT control and 5XFAD control mice. Study 1 (Matthews et al, 2020); Study 2 (Matthews et al, 2019); n = 9-12 per treatment group; **p<0.01, ***p<0.001.

**Supplementary Figure 2**. 5xFAD mice show significantly higher levels of GSL I staining in the cortex and hippocampus versus WT mice. Total GSL I area (%) in the (A) cortex and (B) hippocampus of male and female WT control and 5XFAD control mice. Study 1 (Matthews et al, 2020); Study 2 (Matthews et al, 2019); n = 9-12 per treatment group; **p<0.01, ***p<0.001.

**Supplementary Table 1.** Number of brain samples examined per treatment group.

| | Treatment Group | Aβ | GFAP | GSL I | IL-6 | TNFα | Plasma |
|---|---|---|---|---|---|---|---|
| Female | WT Control H2O | N/A | 12 | 12 | 11 | 9 | N/A |
| | 5xFAD Control H2O | 12 | 12 | 12 | 8 | 11 | 12 |
| | 5xFAD CAW H2O | 12 | 12 | 12 | 9 | 11 | 12 |
| | WT Control Diet | N/A | 11 | 12 | 12 | 12 | N/A |
| | 5xFAD Control Diet | 12 | 12 | 12 | 11 | 11 | 11 |
| | 5xFAD CAW Diet | 11 | 10 | 11 | 11 | 11 | 7 |
| Male | WT Control H2O | N/A | 12 | 12 | 8 | 6 | N/A |
| | 5xFAD Control H2O | 12 | 12 | 12 | 8 | 10 | 11 |
| | 5xFAD CAW H2O | 9 | 10 | 10 | 8 | 9 | 12 |
| | WT Control Diet | N/A | 12 | 12 | 10 | 11 | N/A |
| | 5xFAD Control Diet | 9 | 9 | 9 | 9 | 10 | 11 |
| | 5xFAD CAW Diet | 6 | 6 | 6 | 8 | 10 | 12 |

**Supplementary Table 2.** *Centella asiatica* reference compounds for plasma analysis.

| **Reference Compound** | **CAS Number** | **Supplier** |
|---|---|---|
| *Triterpenes* | | |
| Asiatic acid | 464-92-6 | Sigma Aldrich (Darmstadt, Germany) |
| Asiaticoside | 16830-15-2 | TransMIT (Gießen, Germany) |
| Madecassic acid | 18449-41-7 | TransMIT (Gießen, Germany) |
| Madecassoside | 34540-22-2 | TransMIT (Gießen, Germany) |
| *Caffeoylquinic acids* | | |
| 1,3-dicaffeoylquinic acid | 19870-46-3 | Chromadex (Irvine, CA, USA) |
| 1,5-dicaffeoylquinic acid | 30964-13-7 | Sigma Aldrich (Darmstadt, Germany) |
| Isochlorogenic acid A | 2450-53-5 | TransMIT (Gießen, Germany) |
| Isochlorogenic acid B | 14534-61-3 | TransMIT (Gießen, Germany) |
| Isochlorogenic acid C | 32451-88-0 | Chromadex (Santa Ana, CA, USA) |
| Chlorogenic acid | 327-97-9 | Chromadex (Irvine, CA, USA) |
| Neochlorogenic acid | 906-33-2 | TransMIT (Gießen, Germany) |
| Cryptochlorogenic acid | 905-99-7 | Sigma Aldrich (Darmstadt, Germany) |
| Caffeic acid | 331-39-5 | Sigma Aldrich (Darmstadt, Germany) |
| Dihydrocaffeic acid | 1078-61-1 | Chromadex (Irvine, CA, USA) |
| Ferulic acid | 537-98-4 | Sigma Aldrich (Darmstadt, Germany) |
| Dihydroferulic acid | 1135-23-5 | Toronto Research Chemicals (Toronto, ON, Canada) |
| Isoferulic acid | 537-73-5 | Chromadex (Irvine, CA, USA) |
| Dihydroisoferulic acid | 1135-15-5 | Toronto Research Chemicals (Toronto, ON, Canada) |
| 3-(3-Hydroxyphenyl)propanoic acid | 621-54-5 | Toronto Research Chemicals (Toronto, ON, Canada) |
| *Internal standards* | | |
| $^{13}C_3$-ferulic acid | 1261170-81-3 | Sigma Aldrich (Darmstadt, Germany) |
| Chrysin | 207-549-7 | Honeywell Fluka (Charlotte, NC, USA) |

**Supplementary Table 3.** MS/MS transitions used for compound detection.

| Compound | MS/MS transition (m/z) |
|---|---|
| *Triterpenes* | |
| Asiatic acid | 506/453 |
| Asiaticoside | 976/453; 976/635 |
| Madecassic acid | 522/451 |
| Madecassoside | 992/487; 992/451 |
| *Caffeoylquinic acids* | |
| Mono-CQAs | 353/191 |
| Di-CQAs | 515/353; 515/191 |
| Caffeic acid | 179/135 |
| Dihydrocaffeic acid | 181/109 |
| 3-(3-Hydroxyphenyl)propanoic acid | 165/106 |
| Ferulic acid and isoferulic acid | 193/134 |
| Dihydroferulic acid and dihydroisoferulic acid | 195/136 |
| *Internal standards* | |
| $^{13}C_3$-ferulic acid | 196/136 |
| Chrysin | 255/255 |

**Supplementary Table 4.** Gene expression of inflammatory markers in the deep grey matter. Values represent the fold change for 5xFAD control animals compared to WT control animals.

| | | **Female** | | **Male** | |
|---|---|---|---|---|---|
| **Cytokine** | **Treatment** | **Fold Change** | **SEM** | **Fold Change** | **SEM** |
| IL-6 | Control Water | 1.25 | 0.22 | 1.62 | 0.33 |
| | Control Diet | 1.41 | 0.39 | 1.17 | 0.20 |
| TNFα | Control Water | 2.57*** | 0.25 | 2.99* | 0.81 |
| | Control Diet | 4.03*** | 0.57 | 2.93* | 0.58 |
| IL-1β | Control Water | 4.36*** | 0.67 | 3.07** | 0.66 |
| | Control Diet | 9.42*** | 1.71 | 5.61*** | 1.45 |
| TREM2 | Control Water | 5.27*** | 0.45 | 7.74*** | 1.33 |
| | Control Diet | 7.54*** | 1.59 | 9.10*** | 2.06 |
| AIF1 | Control Water | 2.14*** | 0.10 | 2.51*** | 0.46 |
| | Control Diet | 2.02** | 0.29 | 2.91*** | 0.44 |
| CX3CR1 | Control Water | 1.56* | 0.14 | 1.98* | 0.30 |
| | Control Diet | 1.79* | 0.25 | 2.14** | 0.21 |
| CX3CL1 | Control Water | 0.88 | 0.12 | 0.97 | 0.13 |
| | Control Diet | 0.75 | 0.10 | 0.95 | 0.10 |
| CD36 | Control Water | 1.05 | 0.14 | 1.09 | 0.13 |
| | Control Diet | 0.75 | 0.10 | 1.33 | 0.17 |
| C3AR1 | Control Water | 5.84*** | 0.30 | 5.90*** | 1.24 |
| | Control Diet | 6.86*** | 0.90 | 6.72*** | 1.16 |
| RAGE | Control Water | 1.32 | 0.23 | 1.49 | 0.34 |
| | Control Diet | 1.19 | 0.19 | 1.00 | 0.15 |
| CCR6 | Control Water | 1.23 | 0.31 | 1.30 | 0.51 |
| | Control Diet | 1.63 | 0.44 | 1.07 | 0.25 |
| CD3E | Control Water | 1.89 | 0.31 | 2.01 | 0.39 |
| | Control Diet | 1.76 | 0.25 | 2.76* | 0.50 |

*p<0.05, **p<0.01, ***p<0.001

**References:**


Matthews, D. G., Caruso, M., Magana, A. A., Wright, K. M., Maier, C. S., Stevens, J. F., Gray, N. E., Quinn, J. F. & Soumyanath, A. (2020) Caffeoylquinic acids in centella asiatica reverse cognitive deficits in male 5XFAD Alzheimer's disease model mice. *Nutrients*, 12(11), 1-9.

Matthews, D. G., Caruso, M., Murchison, C. F., Zhu, J. Y., Wright, K. M., Harris, C. J., Gray, N. E., Quinn, J. F. & Soumyanath, A. (2019) Centella asiatica improves memory and promotes antioxidative signaling in 5XFAD mice. *Antioxidants*, 8(12).
