# Supplementary figures and images for: Mode of administration influences plasma levels of active Centella asiatica compounds in 5xFAD mice while markers of neuroinflammation remain unaltered
Source: Front Neurosci. 2024 Mar 25;18:1277626. doi: 10.3389/fnins.2024.1277626 (PMC10999680; doi:10.3389/fnins.2024.1277626)

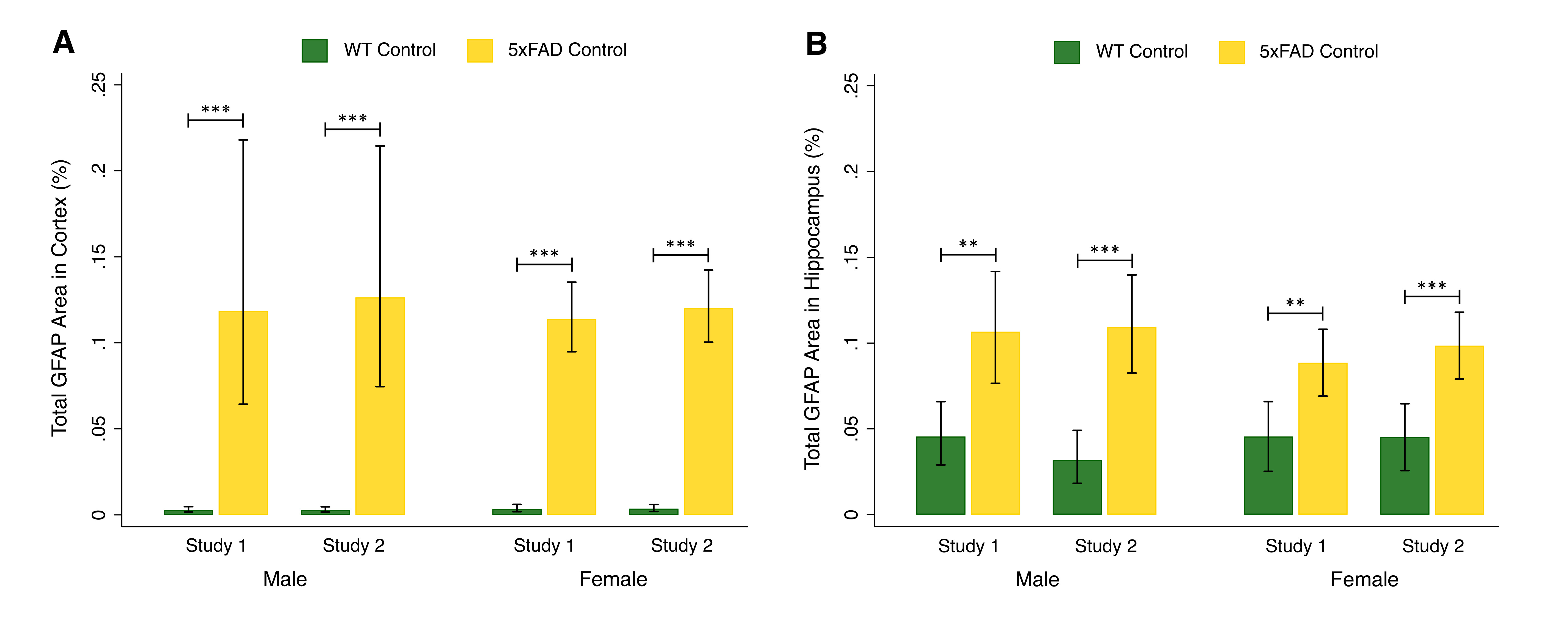

Supplement: Supplementary file 2 [file Image_1.JPEG]

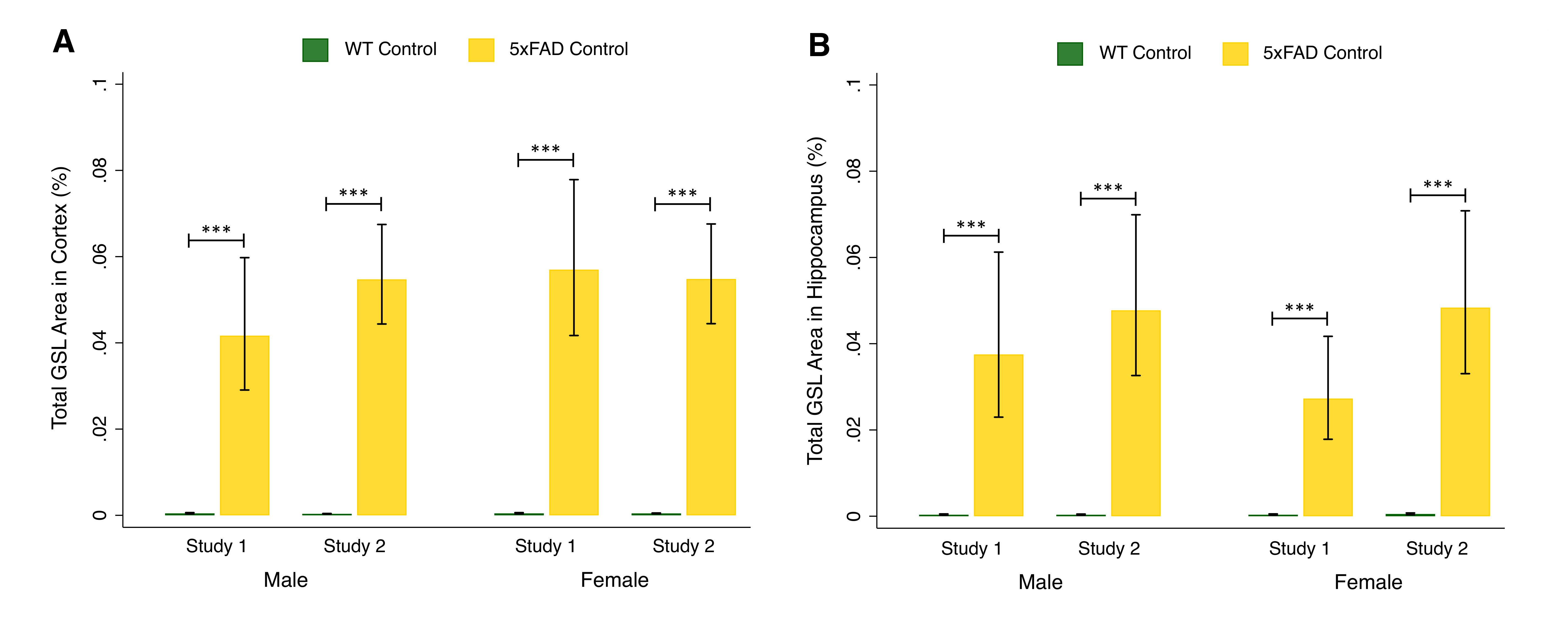

Supplement: Supplementary file 3 [file Image_2.JPEG]
